# Vegetation communities and soil properties along the restoration process of the Jinqianghe mine site in the Qilian Mountains, China

**DOI:** 10.3389/fpls.2024.1358309

**Published:** 2024-04-22

**Authors:** Xiaomei Yang, Qi Feng, Meng Zhu, Jutao Zhang, Linshan Yang, Chengqi Zhang, Zhiyang Wang, Yonglin Feng

**Affiliations:** ^1^ Key Laboratory of Ecohydrology of Inland River Basin, Alax Desert Eco-hydrology Experimental Research Station, Qilian Mountains Eco-Environment Research Center in Gansu Province, Northwest Institute of Eco-Environment and Resources, Chinese Academy of Sciences, Lanzhou, China; ^2^ Technology Innovation Center for Mine Geological Environment Restoration in the Alpine and Arid Regions, Ministry of Natural Resources, Lanzhou, China

**Keywords:** alpine mining area grassland, grassland ecosystem, restoration, plant diversity, community, Qilian Mountains

## Abstract

The study explores the impact of mine grassland restoration on plant communities and soil properties in alpine grasslands, a subject of significant interest due to the observed relationship between grassland changes, plant communities, and soil properties. While prior research has mainly focused on the consequences of grassland degradation on plant diversity and soil characteristics, the specific effects of varying restoration degrees in alpine mining grasslands at the regional scale remain poorly understood. To address this knowledge gap, we established 15 sampling plots (0.5m×0.5m) across five different restoration degrees within alpine mining grasslands in the Qilian Mountains, China. Our objective was to assess the variations in plant diversity and soil properties along these restoration gradients. We conducted comprehensive analyses, encompassing soil properties [soil water content (SWC), available nitrogen (AN), total phosphorus (TP), nitrate nitrogen (NO_3_-N), ammonium nitrogen (NH_4_-N), total nitrogen (TN), available phosphorus (AP), soil organic carbon (SOC), nitrate nitrogen, soil pH, and electrical conductivity (EC)], plant characteristics (height, density, frequency, coverage, and aboveground biomass), and plant diversity indices (Simpson, Shannon-Wiener, Margalef, Dominance, and Evenness indexes). Our findings included the identification and collection of 18 plant species from 11 families and 16 genera across the five restoration degrees: Very Low Restoration Degree (VLRD), Low Restoration Degree (LRD), Moderate Restoration Degree (MRD), High Restoration Degree (HRD), and Natural Grassland (NGL). Notably, species like *Carex duriuscula*, *Cyperus rotundus*, and *Polygonum viviparum* showed signs of recovery. Principal component analysis and Pearson correlation analysis revealed that soil pH, SWC, SOC, NO_3_-N, and AN were the primary environmental factors influencing plant communities. Specifically, soil pH and EC decreased as restoration levels increased, while SWC, AN, TP, NH_4_-N, TN, AP, SOC, and NO_3_-N exhibited a gradual increase with greater restoration efforts. Furthermore, the HRD plant community demonstrated similarities to the NGL, indicating the most effective natural recovery. In conclusion, our study provides valuable insights into the responses of plant community characteristics, plant diversity, and soil properties across varying restoration degrees to environmental factors. It also elucidates the characteristics of plant communities along recovery gradients in alpine grasslands.

## 1 Introduction

Alpine grasslands in Northwest China serve as a critical ecological buffer, offering vital ecosystem functions, including livestock grazing, landscape aesthetics, and vegetation production (Wang et al., 2018). However, the alpine grassland ecosystem is notably sensitive to human activities and climate fluctuations, rendering it highly susceptible to issues like grassland degradation, soil contamination, and declining vegetation cover (Lei et al., 2020; Wang and Ali, 2021). Once an ecological imbalance occurs, ecosystem recovery becomes a formidable challenge (Dudley et al., 2020). Northwest China boasts abundant mineral resources, yet imprudent or excessive mining practices can trigger a range of ecological problems, including soil quality deterioration due to human interventions (Wu et al., 2021). Consequently, this contributes to the degradation of mining sites. Achieving equilibrium between the alpine grassland ecosystem and mining grassland ecosystems has emerged as a significant scientific endeavor (Xie et al., 2017).

Plant communities and soil properties share a close relationship, with soil properties serving as a determinant of plant composition (Jochum et al., 2020). Human activities, climate conditions, and soil properties collectively influence the development of alpine grassland ecosystems. Mining, as a significant factor, contributes to grassland degradation and alters plant community composition (Wiegand et al., 2007; Chang et al., 2015). However, it’s important to note that variations in soil properties primarily define the plant community in mining grasslands (Zhang et al., 2018; Gao et al., 2021). Although prior research has acknowledged the impact of excessive mining on alpine grassland ecosystems, there remains a gap in understanding the mechanisms behind the changes in grassland restoration levels (Guo et al., 2021; Huang et al., 2021a; Castro et al., 2023). In particular, studying how the plant community evolves can shed light on grassland restoration mechanisms (Kang et al., 2020). Plant community characteristics can provide insights into both plant diversity, encompassing variety and function, and how they respond to different restoration levels (Harrison et al., 2020). Examining the diversity of plant species and their functions across various restoration levels is a compelling avenue of inquiry. While previous studies have predominantly focused on individual plant communities or soil properties at different restoration levels (Feyissa et al., 2021; Stokes et al., 2021), there is a limited body of research on how plant communities and plant diversity change across these levels (Zhou et al., 2019; Schmid et al., 2021). Quantifying plant community and soil properties at different restoration levels can enhance our understanding of how plant communities and plant diversity evolve in alpine grasslands.

The Qilian Mountains National Nature Reserve is a crucial national ecological sanctuary in China, meticulously preserved and protected. It plays an indispensable role in shaping and efforts have been made to enhance the ecological environment in the Western region (Feng et al., 2019). However, mining activities in the Qilian Mountains Reserve have led to ecological damage, resulting in the degradation of the original grassland and soil erosion (Kong et al., 2021). Presently, numerous researchers have been actively involved in managing the ecological environment of mining areas and implementing scientific and rational approaches for the restoration of degraded grassland in these regions. The ecological balance of moderate restoration of its productivity and economic benefits is particularly important (Chen et al., 2020). The mining area in the Qilian Mountains in the Tianzhu region of China has expanded rapidly in recent years, and the largest open mine in the Gansu Province is located in this region. The ecological and environmental damage to the nature reserve has aroused considerable attention. However, there have been few studies on how plant community features and soil properties changes in mining areas have affected the mining grasslands (Chen et al., 2022).

While some studies have explored the restoration of mining areas, there is a notable gap in understanding how restored plant community features vary. Additionally, there has been limited research on plant community features and soil properties across different restoration levels in alpine grasslands (Lorite et al., 2021; Siebert et al., 2021). To address this knowledge gap, our study investigates plant communities and soil properties at various restoration levels within the primary gold mining area of alpine grasslands in the Qilian Mountains, China. We hypothesize that plant communities, plant diversity, and soil properties strongly influence the outcomes at different restoration levels in alpine grasslands. Specifically, our study seeks to achieve the following objectives: (1) Assess the restoration outcomes of alpine mining grassland ecosystems compared to natural grasslands; (2) Investigate how plant communities, plant diversity, functional diversity, and soil properties change at various levels of restoration within the mining area. The findings from this study aim to provide valuable insights into how different restoration levels respond to environmental factors. Additionally, our research intends to establish a scientific foundation for the rational restoration of alpine grassland resources in mining areas.

## 2 Materials and methods

### 2.1 Study area description

**Figure 1:**
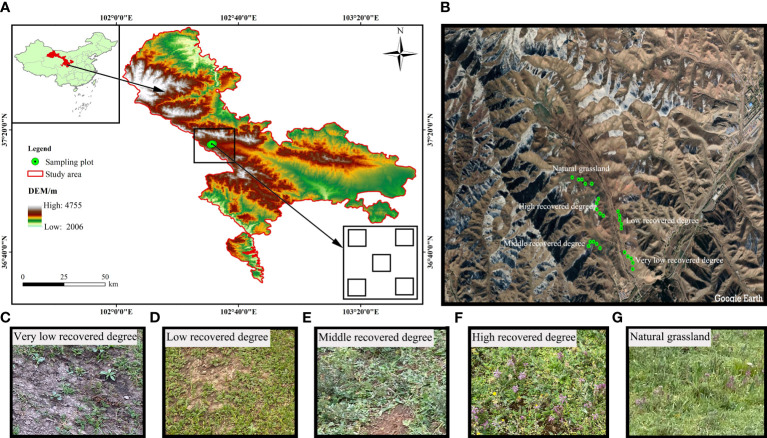
Location of the study area on the Chinese Qilian Mountains **(A, B)** and VLRD, very low recovered degree **(C)**, and LRD, low recovered degree **(D)** and MRD, middle recovered degree **(E)** and HRD, high recovered degree **(F)** and NGL, CK natural grassland **(G)**.

The Jinqianghe mining area is located in the part of Daiqian village, Zhuaxixiulong Town, Wuwei City, Gansu Province. The geographical coordinates are 37°25’30”-37°25’63”N, 102°51’48”-102°57’82”E (**Figures 1A, B**), and the altitude is 3267-3323m. This area is located at the junction of Gansu and Qinghai provinces, Zhuanglang River Jinqiang River source of the typical gold mine restoration demonstration area, in Qilian Mountains National Nature Reserve. Soil types in the project area are mainly grassland meadow soils, followed by large black soils and black hemp soils. There is no treatment of the original soil because the sampling places are in the meadow of the Jinqianghe mining area. Jinqianghe upstream alluvial gold mining area is in the headwaters of the Jinqianghe, the source of the Zhuanglang River, the overall topography of the northwest high southeast low, the topography of the undulating changes, elevation generally 3190~4100 m, the relative height difference of the mountains are more than 500 m, the slope of the mountain slope is generally 30~60°, the local section of the slope is greater than 70°. The valley of Jinqianghe is generally 200m~300m wide, and the widest part can reach 1000m, with the first and second terraces developed and third terraces sporadically developed.

The ecological environment was fragile before the mine was restored, the land was barren and the degree of soil erosion was severe, clearly improving the ecological environment after the mine restoration. The region has an alpine semi-arid climate, with seasonal changes of temperature in the region being obvious, with the highest temperature being in July, and the lowest temperature being in January, and a huge diurnal temperature difference. The average annual temperature is 4.8°C. The average annual rainfall and potential evaporation are 650mm and 1400mm, separately, and the rainy season is mainly focused on the June-September. The distinctive feature is clear vertical zoning, low temperatures, high precipitation (more topographic rain) and short frost-free periods. The Northwest winds are prevalent in the area, with the annual average wind speed is 2.1m/s. The main soil type is grassland meadow soil, followed by big black soil, black hemp soil, and other soil types. Before restoration, due to the serious damage to the ecological environment caused by mining activities, a large amount of dumped soil and slag were piled up randomly, the soil was pressed and dug up, and the vegetation cover was extremely low, mainly through the natural restoration method of slag heap leveling, sowing grass seeds and fencing protection. With the expansion of the scale within the area of mining, the waste slag after mining destroyed the vegetation and degraded the vegetation on both sides of the river valley and the ditch, and the vegetation coverage on both sides of the river valley and the ditch before treatment was lower, the mine restoration from 2018 by the same restoration method of natural restoration (Yang et al., 2023a). The primary methods for natural restoration include the leveling of slag heaps, sowing of grass seeds, fence protection, and the facilitation of natural vegetation recovery. Removal of residue piles, backfilling of mining pits, restoration of vegetation in pressure-occupied areas and backfilled areas, and restoration of topography and ecological environment to the greatest extent possible. The approach for restoring succession sequences is determined according to the “Soil Erosion Classification and Grading Standard” (SL190-2007) of China. In accordance with the coverage of natural restoration, we used the temporal dynamics of restoration process by replacing the time scale with space, for the investigation, representative grassland restoration in the Qilian Mountains Tianzhu Jinqianghe gold mine was selected. The study encompassed five different levels of grassland recovery based on plant coverage, classified as follows: Very Low Recovered Degree (VLRD), Low Recovered Degree (LRD), Middle Recovered Degree (MRD), High Recovered Degree (HRD), and a reference of Natural Grassland (NGL), with Natural Grassland (NGL) serving as the control (CK), as indicated in **Table 1**. The vegetation primarily consists of *Cyperus rotundus, Polygonum viviparum, Oxytropis ochrocephala* and *Elymus nutans*, and the animal husbandry industry is the leading industry.

**Table 1:** Sample plots settings.

	VLRD	LRD	MRD	HRD	NGL
**Geography coordinate**	N37^°^25’30” E102^°^51’48”	N37^°^25’67” E102^°^52’24”	N37^°^25’51” E102^°^52’78”	N37^°^25’61” E102^°^52’82”	N37^°^25’62” E102^°^52’18”
**Altitude/m**	3320	3294	3286	3292	3295
**Plant coverage**	<10%	20%-40%	40%-60%	>85%	>85%
**Percentage of bare land**	>50%	25%-40%	10%-25%	<5%	<5%
**Soil characteristics**	Comparatively low level	Low level	Middle level	High level	Nature level
**Distance from mine development site**	Nearest	Near	Middle	Far	Far

VLRD, very low recovered degree; LRD, low recovered degree; MRD, middle recovered degree; HRD, high recovered degree; NGL, CK natural grassland.

### 2.2 Sample collection and testing

This study primarily focuses on investigating various soil parameters, including pH, electrical conductivity (EC), soil water content (SWC), soil organic carbon (SOC), total nitrogen (TN), nitrate nitrogen (NO_3_-N), ammonium nitrogen (NH_4_-N), available nitrogen (AN), total phosphorus (TP), and available phosphorus (AP). The data collection was conducted in August 2022. Three study sites with similar geographic conditions, vegetation composition, and several years of restoration management were selected. To ensure consistency and representativeness in vegetation and soil sampling, we established three large quadrats within each of the five different restoration grassland areas. Within each large quadrat, five small sampling plots (measuring 50×50 cm) were randomly chosen in the mining area for sample collection, with precise GPS coordinates recorded for accurate positioning (**Figure 1**). Differences in vegetation restoration were observed, particularly in areas closer to the road, due to varying distances from the mine’s pollution source. Despite employing the same restoration method, disparities in vegetation recovery persisted. We measured plant circumference, plant height using a steel tape measure, and recorded the plant altitude, longitude, and latitude for each location using GPS devices. Plant community characteristics, including plant frequency, plant density, plant coverage, and plant composition, were documented (Gao et al., 2021). For each sampling plot, we collected soil samples (at depths of 0-20 cm and 20-40 cm) from five points, which were subsequently mixed thoroughly to create a composite sample. These soil samples were transported to the laboratory, where they were naturally air-dried, and any rocks, plant roots, or debris were removed. Afterward, the samples were ground, passed through a 120-mesh sieve, properly labeled, and prepared for testing.

The SWC was measured by the drying method. The soil pH, EC, SOC, TN, NO_3_-N, NH_4_-N, AN, TP and AP were determined by the electrode potential (Pansu and Gautheyrou, 2006), conductivity meter, dichromate oxidation, element analyzer, Interval-flow analysis, Flow-Injection, alkaline solution-diffusion, Sommers-Nelson, and molybdenum antimony anti-colorimetric methods (Pei et al., 2021), respectively.

### 2.3 Data processing methods

The indexes of species diversity and functional diversity within the plant characteristics were examined to explore the changing of plant diversity across restoration gradients. Community Importance Value (IV) serves as a crucial criterion for distinguishing various communities and efficiently determining the major components of each community. The calculation method for the Importance Value (IV) is as follows:

IV = (Relative Height + Relative Cover + Relative Density)/3 (Zhang, 2004).

Species diversity is evaluated through factors like species richness, diversity indices, and evenness. This is expressed using measures such as degree and dominance degree (Ma, 1994). The calculation is as follows:


(1) $$\text{H}=-\sum {\text{P}}_{\text{i}}\ln{\text{P}}_{\text{i}}$$

(2) Ma=(S−1)/lnN

(3) $$\text{D}=1-\sum {\text{P}}_{\text{i}}^{2}$$

(4) Eve=D/(1−1/S)

(5) $$D_{o}=\sum {\text{P}}_{\text{i}}^{2}$$

(6) $${\text{P}}_{\text{i}}={\text{N}}_{\text{i}}/\text{N}$$

In the preceding formula, the symbols are defined as follows: H denotes the Shannon-Wiener species diversity index (1), Ma represents the Margalef richness index (2), D stands for the Simpson diversity index (3), Eve corresponds to the Evenness index (4), Do signifies the Dominance index (5), Pi denotes the species “i” important value ratio (6), N is the sum of the important values of plants in the transect, Ni represents the important value of plant “i” in the plot, and S is the total number of species in the plot.

### 2.4 Statistical analysis

Soil physical and chemical properties for data analysis were obtained from average measurements at depths of 0-20 cm and 20-40 cm per sample. Plant biomass values were determined for the underlying biomass. To assess differences among restored grassland communities, one-way analysis of variance (ANOVA) was applied. Two-tailed relationships between plant community and soil properties were analyzed using Spearman correlation coefficients in SPSS version 25.0 (IBM, Chicago, USA). Principal Component Analysis (PCA) in CANOCO 5.0 (ter Braak and Smilauer, 2012) was employed to explore diversity variations along recovery degrees for enhanced plant distribution pattern assessment. Important value and diversity index for each herb layer plant were computed using Microsoft Excel 2010. Correlation analysis and graph plotting were performed in Origin 9.0 (Origin Lab Corporation, Northampton, MA, USA), while bar charts and additional data analysis were carried out in Sigmaplot 10.0 (Sigmaplot Lab Corporation, Northampton, MA, USA).

## 3 Results

### 3.1 Characteristics of plants at various restoration degrees

**Figure 2:**
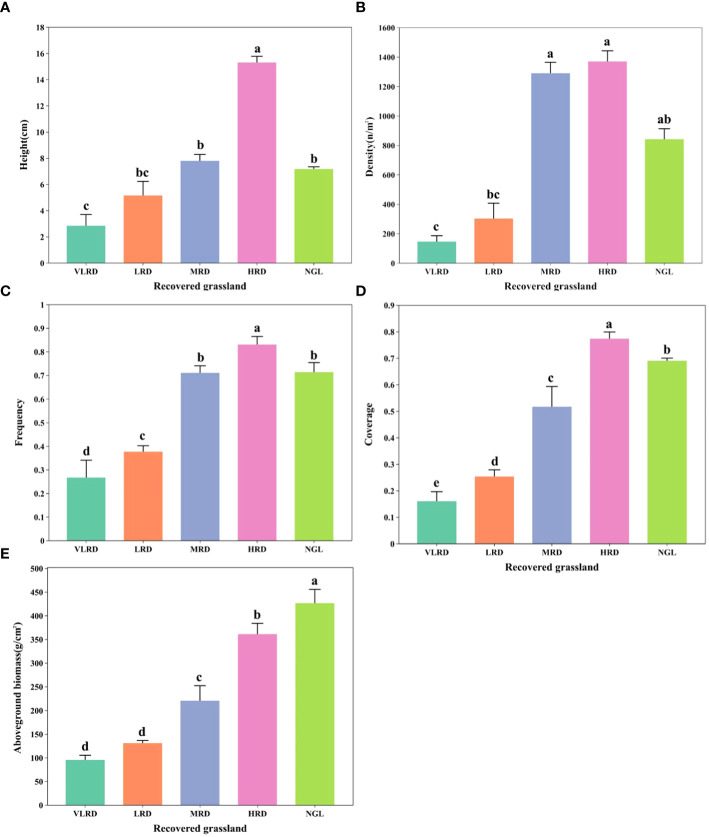
Illustrates the vegetation characteristics of alpine grassland at various stages of restoration, with five key attributes: **(A)** Height, **(B)** Density, **(C)** Frequency, **(D)** Coverage, and **(E)** Aboveground biomass. The different restoration levels are represented by the following abbreviations: VLRD (Very Low Recovery Degree), LRD (Low Recovery Degree), MRD (Middle Recovery Degree), HRD (High Recovery Degree), and NGL (CK Natural Grassland). Significant differences at the 0.05 level are denoted by different letters above error bars between the treatments.

The plant characteristics of alpine mining grassland communities exhibited significant variations across different levels of restoration (**Figure 2**). Vegetation features displayed notable distinctions (*P*<0.05) among the various restoration levels, with the ranking being NGL > HRD > MRD > LRD > VLRD. The height of HRD was 1.66, 2.52, and 4.58 times that of MRD, LRD, and VLRD, respectively (**Figure 2A**). Additionally, its density exceeded that of MRD, LRD, and VLRD by 1.06, 4.54, and 9.42 times, as shown in **Figure 2B**. The frequency of HRD surpassed that of MRD, LRD, and VLRD by 1.17, 2.18, and 3.07 times (**Figure 2C**), while its coverage was 1.48, 3.08, and 4.81 times greater than MRD, LRD, and VLRD (**Figure 2D**), respectively. Moreover, the aboveground biomass of HRD was 1.64, 2.76, and 3.79 times that of MRD, LRD, and VLRD (**Figure 2E**). Notably, natural restoration methods did not achieve the level of NGL (CK).

### 3.2 The composition of plant families, genera, and species varies across different restoration degrees

**Table 2** presents the taxonomic structure of alpine mining grassland at various stages of restoration. It is evident that the order of species composition varies with different restoration degrees, with HRD > MRD > LRD > VLRD. The total number of individual genera in the various community types aligns with the species composition pattern. According to the statistical data from our sample survey, the study plot contained 18 herbaceous plant species, which were distributed across 11 families and 16 genera. Among these, Asteraceae had the highest species diversity with 6 genera and 6 species. This was followed by Leguminosae and Cyperaceae, each with 2 genera and 2 species, while Rosaceae, Graminaceae, Ranunculus, Polygonum, Chenopodium, Plantago, Apiaceae, and Geraniaceae each had 1 genus and 1 species. This demonstrates the simplicity of the plant genus and species structure, with a relatively dispersed distribution of plant families. Across all samples, three species—*Carex duriuscula*, *Cyperus rotundus*, and *Polygonum viviparum*—were consistently present. Plant species from five different recovery degrees belonged to the Leguminosae, Asteraceae, Compositae, Rosaceae, and Cyperaceae families, and these families exhibited a high degree of representation. Among them, these five major plant families constituted 67% of the total plant species in the HRD community, 55% in the MRD, 86% in the LRD, and 50% in the VLRD. This pattern is largely attributed to the adaptability of Leguminosae and Graminaceae to the challenging environmental conditions characterized by low temperatures and drought in alpine grassland mining areas. Notably, the plant composition in the NGL (CK Natural Grassland) demonstrated the highest degree of diversity in alpine mining grassland areas.

**Table 2:** Composition of dominant plant families, genera, and species at various levels of recovery.

Recovered degree	Total families	Total genera	Total species	The species distribution of common families						
				Legumino sae	Compositae	Compositae	Rosaceae	Cyperaceae	Total	Rate in total%
VLRD	3	4	4	0	0	0	0	2	2	50%
LRD	6	7	7	1	1	1	1	2	6	86%
MRD	10	11	11	1	1	1	1	2	6	55%
HRD	9	12	12	1	3	1	1	2	8	67%
NGL	11	13	13	1	2	1	1	2	7	54%

VLRD, very low recovered degree; LRD, low recovered degree; MRD, middle recovered degree; HRD, high recovered degree; NGL, CK natural grassland.

### 3.3 Life-form structure characteristics of plant communities

The life-form structure characteristics of plant communities at various restoration levels in alpine mining grassland are shown in **Table 3**. The life-form classification follows the Whitaker growth type system, which is based on the degree of stem lignification in the community, as described by Yang et al. (2023a). This study categorizes the herbaceous plant community into two groups: perennial plants and annual herbaceous plants. Among the life-form structure characteristics, perennial herbaceous species predominate, with 12 species distributed across 9 families and 12 genera, while annual herbaceous species are less dominant, consisting of 4 species from 3 families and 4 genera. Perennial herbaceous species account for 75% of the total species, while annual herbaceous species make up the remaining 25% (**Table 3**). There is a significant disparity in the abundance of perennial and annual herbaceous species among various restoration levels in alpine mining grassland, with perennial herbaceous species being abundant and annual herbaceous species relatively scarce. Particularly in the NGL, perennial herbaceous species dominate at 97%, and they also constitute a significant portion in HRD, MRD, LRD, and VLRD, with proportions of 88%, 77%, 96%, and 100%, respectively. Conversely, MRD has the largest proportion of annual herbaceous species, amounting to 23%. It’s worth mentioning that the presence of perennial herbaceous species significantly influences the structure, system function, and stability of the grassland community. Although the species structure of NGL grassland is slightly higher than that of HRD, HRD exhibits the most stable species community structure, followed by MRD, LRD, and VLRD grasslands.

**Table 3:** Composition and quantitative traits of communities at different recovery stages.

Life type	Species	Species number in different recovered degrees of alpine grassland				
		VLRD	LRD	MRD	HRD	NGL
Perennial herb	*Cyperus rotundus*	5	22	74	103	71
	*Carex duriuscula*	9	22	47	71	12
	*Taraxacum mongolicum*	—	—	6	3	7
	*Polygonum viviparum*	5	13	10	73	45
	*Oxytropis ochrocephala*	—	3	48	52	54
	*Elymus nutans*	—	2	—	10	12
	*Potentilla chinensis*	—	—	10	—	14
	*Plantago Asiatica*	—	—	5	15	35
	*Daucus carota*	21	16	13	6	21
	*Cirsium arvense*	—	—	—	—	11
	*Anaphalis lactea Maxim*	—	—	3	2	—
	*Geranium pratense*	—	—	—	—	5
	subtotal	40	78	216	335	287
	subtotal%	100%	96%	77%	89%	97%
Annual herb	*Chenopodium glaucum*	—	—	64	43	5
	*Sonchus oleraceus*	—	—	—	1	—
	*Heteropappus hispidus*	—	3	—	—	—
	*Ipomoea nil*	—	—	—	—	3
	subtotal	0	3	64	44	8
	subtotal%	0%	4%	23%	12%	3%
	Total	40	81	280	378	295
	Total%	100%	100%	100%	100%	100%

VLRD, very low recovered degree; LRD, low recovered degree; MRD, middle recovered degree; HRD, high recovered degree; NGL, CK natural grassland.

### 3.4 Plant community value of different restoration degrees

Perennial herbaceous plants occupy a dominant position within various communities, playing a crucial role in shaping community structure, ecosystem function, and overall stability. The importance of plant communities at different restoration levels in alpine grassland is detailed in **Table 4**. In HRD, LRD, and VLRD, *Carex duriuscula* stands out as the dominant species with importance values of 18.53, 27.33, and 39.22, respectively. The sub-dominant species in these communities are *Cyperus rotundus* and *Polygonum viviparum*. Notably, the importance values for HRD are 16.70 and 15.64 for Cyperus rotundus and Polygonum viviparum, respectively. In MRD, the dominant species is *Carex duriuscula* with an importance value of 21.40, while the sub-dominant species is represented by *Cyperus rotundus* and *Polygonum viviparum* with respective importance values of 4.53. In HRD, the primary companion species include *Chenopodium glaucum* and *Sonchus oleraceus*, boasting importance values of 7.71 and 1.11, respectively. For VLRD, *Carex duriuscula* and *Daucus carota* are the dominant species with importance values of 39.22 and 27.11. The importance values of other companion species are relatively similar and remain below 10. When we compare alpine grassland herbaceous communities across four restoration-degree mining areas, it becomes evident that the importance values of perennial herbs in these communities consistently exceed 10. This underscores the significant role played by perennial herbs in the recovery of alpine grassland mining areas, with HRD approaching the level of NGL, suggesting that HRD has a highly favorable recovery status.

**Table 4:** Important value of dominant and sub-dominant species under different recovered stages.

Recovered stage	VLRD	Important value	LRD	Important value	MRD	Important value	HRD	Important value	NGL	Important value
Dominant species	*Carex duriuscula*	39.22	*Carex duriuscula*	27.33	Cyperus rotundus	21.40	*Carex duriuscula*	18.53	*Oxytropis ochrocephala*	18.95
	*Daucus carota*	27.11	*Potentilla chinensis*	26.00	*Carex duriuscula*	18.90	Cyperus rotundus	16.70	Cyperus rotundus	16.92
Sub-dominant	Cyperus rotundus	23.69	Cyperus rotundus	24.13	*Chenopodium glaucum*	15.48	Polygonum viviparum	15.64	*Plantago asiatica*	11.62
			*Oxytropis ochrocephala*	8.03	*Oxytropis ochrocephala*	14.81	*Oxytropis ochrocephala*	14.43	*Carex duriuscula*	10.79
Main Companion specie	Polygonum viviparum	9.98	Polygonum viviparum	7.61	Polygonum viviparum	8.10	*Chenopodium glaucum*	7.71	*Daucus carota*	9.94
			*Elymus nutans*	4.27	*Daucus carota*	5.82	*Potentilla chinensis*	6.96	*Elymus nutans*	6.74
			*Heteropappus hispidus*	2.62	*Taraxacum mongolicum*	5.49	*Plantago asiatica*	6.02	*Taraxacum mongolicum*	6.18
					*Potentilla chinensis*	4.53	*Daucus carota*	4.28	Polygonum viviparum	6.10
					*Plantago asiatica*	3.63	*Taraxacum mongolicum*	3.75	*Potentilla chinensis*	3.68
					*Ipomoea nil*	0.97	*Elymus nutans*	2.67	*Chenopodium glaucum*	3.28
					*Elymus nutans*	0.88	*Anaphalis lactea Maxim*	1.34	*Geranium pratense*	2.75
							*Sonchus oleraceus*	1.11	*Ipomoea nil*	2.20

VLRD, very low recovered degree; LRD, low recovered degree; MRD, middle recovered degree; HRD, high recovered degree; NGL, CK natural grassland.

### 3.5 Variations in community life structure characteristics across different restoration levels

In terms of community life structure, the characteristics of different restoration degrees in alpine mining grassland exhibited a consistent trend between the two life forms—perennial herbaceous plants were significantly dominant, collectively representing more than 83.56% of the total importance values, while annual herbaceous plants had a smaller share (**Table 5**). Across the various restoration levels, perennial herbaceous plants consistently held higher importance values than annual herbaceous plants. Notably, alpine grasslands with NGL, HRD, and LRD had relatively high importance values for perennial herbaceous plants. In addition, alpine grasslands with HRD and MRD displayed relatively high importance values for annual herbaceous plants. Perennial herbaceous species in these communities primarily belonged to the Graminaceae, Asteraceae, Leguminosae, Rosaceae, and Cyperaceae families. On the other hand, annual herbs were primarily represented by the following four species: *Sonchus oleraceus*, *Chenopodium glaucum*, *Heteropappus hispidus*, and *Ipomoea nil*. When examining alpine grasslands with different restoration levels, HRD featured the highest number of plant species, mainly composed of perennial herbaceous plants, while VLRD lacked annual herbaceous species. Within these restoration grassland communities, perennial herbaceous plants were predominantly *Carex duriuscula*, *Cyperus rotundus*, *Oxytropis ochrocephala*, *Potentilla chinensis*, and *Polygonum viviparum*. Annual herbaceous plants primarily included *Chenopodium glaucum*, *Sonchus oleraceus*, *Heteropappus hispidus*, and *Ipomoea nil*.

**Table 5:** Relative importance of life forms in grassland communities at various recovery degrees (%).

Recovered degree	Important value	
	Perennial herbaceous plant	Annual herbaceous plant
VLRD	100.00	0.00
LRD	97.38	2.62
MRD	83.56	16.44
HRD	90.31	9.69
NGL	94.51	5.49
Total	465.76	34.24

VLRD, very low recovered degree; LRD, low recovered degree; MRD, middle recovered degree; HRD, high recovered degree; NGL, CK natural grassland.

### 3.6 Variations in plant diversity across restoration levels

**Figure 3:**
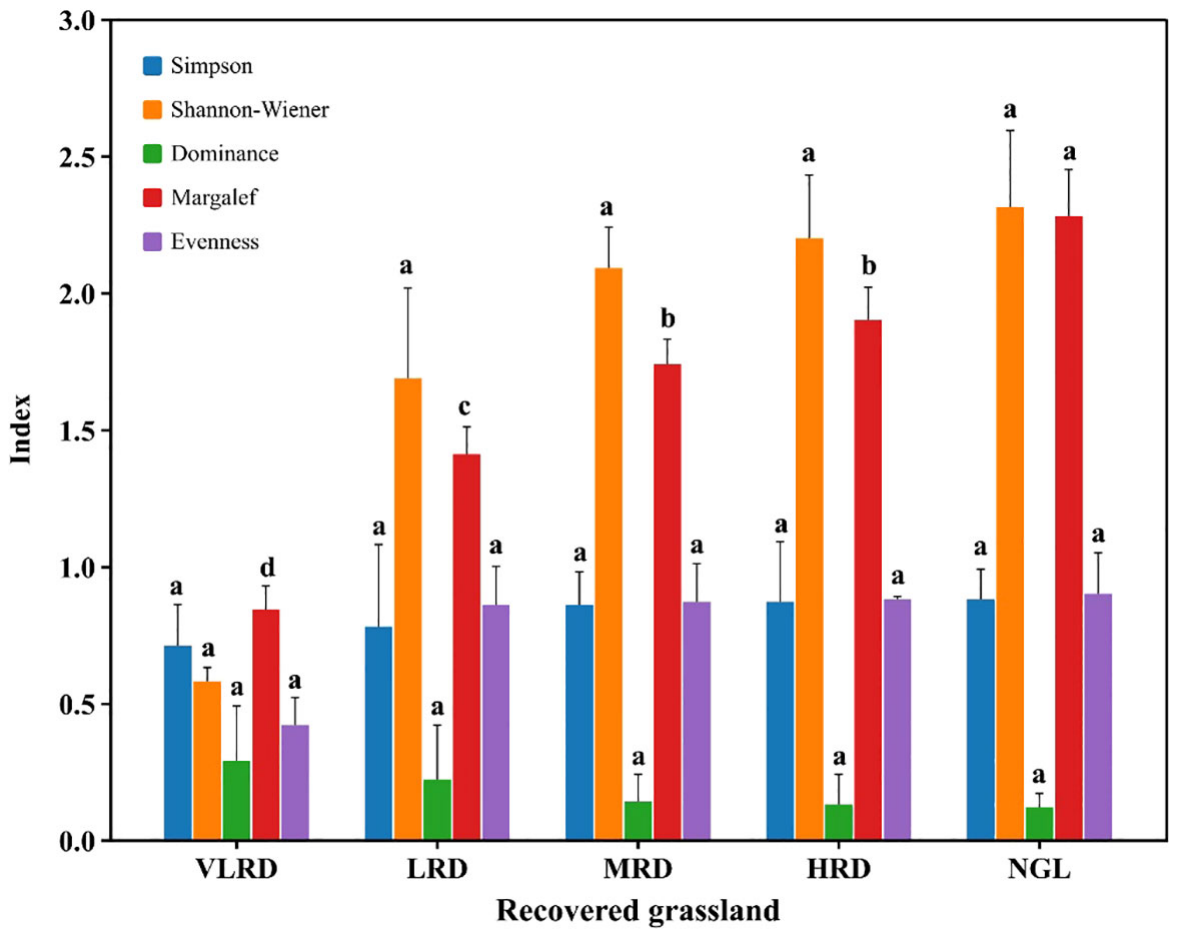
Variation in plant diversity in alpine meadows under different restoration degrees. Different colors represents Simpson and Shannon-Wiener Indices, Dominance, Margalef, and Evenness Indexes. VLRD stands for Very Low Recovered Degree, LRD for Low Recovered Degree, MRD for Middle Recovered Degree, HRD for High Recovered Degree, and NGL for CK Natural Grassland. Different letters above error bars between treatments indicate statistically significant differences at the 0.05 level.

The variations in plant diversity across different restoration levels in alpine grassland is displayed in **Figure 3**. The Simpson index indicates that HRD and MRD have higher diversity indices compared to VLRD, with no statistically significant differences between HRD and MRD, and VLRD (*P*>0.05). Their respective values are 0.87, 0.86, and 0.71. Likewise, the Shannon-Wiener index is higher in HRD and MRD compared to VLRD, with no significant differences between HRD and MRD, and LRD (*P*>0.05). The values are 2.20, 2.09, and 1.69. The Margalef species richness index in HRD is significantly greater than in LRD and VLRD (*P*<0.05). HRD boasts the highest species richness with 12 species, followed by MRD with 11 species, LRD with 7 species, and a minimum of 4 species in VLRD. HRD’s composition closely resembles that of NGL. The Dominance index does not exhibit significant differences among different restoration levels in alpine grassland (*P*>0.05). However, the Dominance index in VLRD is higher than in other restoration levels, with a value of 0.29. Regarding the Evenness index, HRD and MRD surpass LRD, but no significant differences exist between HRD and MRD, and LRD (*P*>0.05), the values were 0.88, 0.87, and 0.86, respectively.

### 3.7 Statistical overview of soil properties

**Figure 4:**
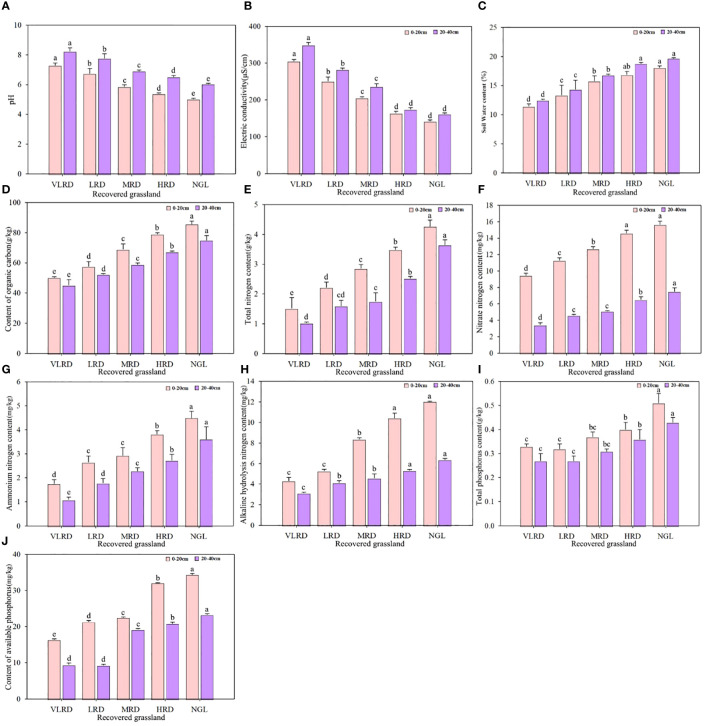
Soil properties of alpine meadows, including pH **(A)**, EC **(B)**, SWC **(C)**, SOC **(D)**, TN **(E)**, NO_3_-N **(F)**, NH_4_-N **(G)**, AN **(H)**, TP **(I)**, and AP **(J)**, Under Different Restoration Degrees. VLRD, very low recovered degree; LRD, low recovered degree; MRD, middle recovered degree; HRD, high recovered degree; NGL, CK natural grassland. Different letters above error bars between treatments indicate significant differences at the 0.05 level.

Soil properties exhibited significant variations across different recovered grasslands (**Figure 4**). Specifically, soil pH, electrical conductivity (EC), and soil water content (SWC) at the 20-40 cm soil depth were significantly higher than those at the 0-20 cm depth (*P*< 0.05). Moreover, soil pH and EC showed a gradual decrease with increasing restoration levels, while soil SWC increased with higher restoration levels. When comparing different restoration levels, the soil pH in VLRD and LRD grasslands at the 0-20 cm soil depth was 44.84% and 33.93% higher, respectively, than that in the NGL. At the 20-40 cm soil depth, the soil pH in VLRD, LRD, MRD, and HRD grasslands was 36.20%, 28.43%, 14.38%, and 7.77% higher, respectively, than in the NGL. Similar trends were observed for soil EC and SWC. Additionally, the HRD exhibited recovery effects that were similar to those of the NGL, indicating the most successful natural recovery. Soil properties, including soil organic carbon (SOC), total nitrogen (TN), nitrate nitrogen (NO_3_-N), ammonium nitrogen (NH_4_-N), available nitrogen (AN), total phosphorus (TP), and available phosphorus (AP), were significantly higher at the 0-20 cm soil depth compared to the 20-40 cm depth (*P*< 0.05). In HRD grassland, SOC, TN, NO_3_-N, NH_4_-N, AN, TP, and AP were significantly higher than in VLRD grassland (*P*< 0.05). These trends gradually increased with higher restoration levels at 10-20 cm and 20-40 cm soil depths. Once again, the HRD demonstrated recovery effects similar to the NGL, signifying the most successful natural recovery.

### 3.8 The connection between plant community diversity and soil properties

**Figure 5:**
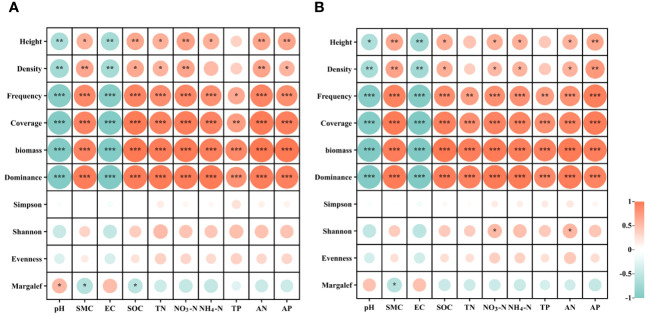
Correlations between plant community diversity and soil physiochemical properties in recovered alpine grassland at soil depths of 0-20 cm **(A)** and 20-40 cm **(B)**. pH, soil pH; SWC, soil water content; EC, soil electric conductivity; SOC, soil organic carbon; AP, soil available phosphorus; TP, soil total phosphorus; TN, soil total nitrogen; NO3-N, soil nitrate nitrogen; NH_4_-N, soil ammonium nitrogen; AN, soil alkaline nitrogen. * indicates significant correlation (p < 0.05), ** indicates extremely significant correlation (p < 0.01), *** indicates extremely significant correlation (p < 0.001).

Pearson correlation analysis was employed to investigate the relationships between plant community diversity and soil properties (**Figure 5**). The results show that, at the 0-20 cm soil depths, soil properties including soil water content (SWC), available nitrogen (AN), total phosphorus (TP), ammonium nitrogen (NH_4_-N), total nitrogen (TN), available phosphorus (AP), soil organic carbon (SOC), nitrate nitrogen (NO_3_-N), pH, and electrical conductivity (EC) exhibited a significant and positive correlation with plant frequency, coverage, and biomass (*P*< 0.01). However, these soil properties were significantly and negatively correlated with plant community diversity indices (Simpson, Shannon, Evenness, and Margalef indices) (*P* > 0.05). Furthermore, soil SOC exhibited a significant positive correlation with plant height (R = 0.65, *P*< 0.01) (**Figure 5A**). At the 20-40 cm soil depths, SWC, AN, TP, NH_4_-N, TN, AP, SOC, NO_3_-N, pH, and EC showed significant and positive correlations with plant frequency, coverage, and biomass (*P<* 0.01). However, these soil properties at 20-40 cm depths were significantly and negatively correlated with plant community diversity indices (*P* > 0.05). Additionally, soil AP demonstrated a significant positive correlation with plant height (R = 0.66, *P*< 0.01) (**Figure 5B**).

**Figure 6:**
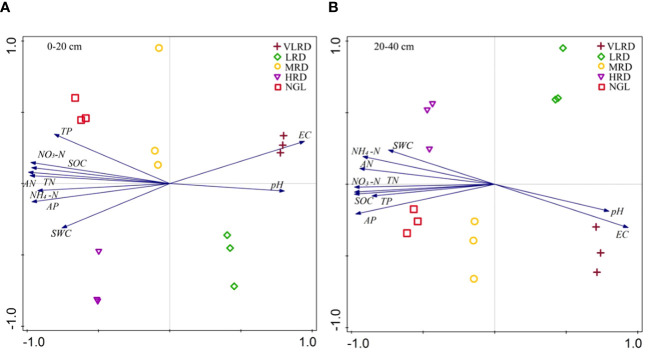
The principal component analysis (PCA) of soil physiochemical properties in alpine recovered grassland at soil depths of 0-20 cm **(A)** and 20-40 cm **(B)** was depicted. In the PCA plot, arrows indicate the direction and strength of the associated soil physiochemical indices, with the length of the arrows reflecting the degree of correlation with the variables. Each graph represents the recovered grassland for each sample (three replicates per sample, 1–3). The data are derived from three replicates (1–3) per sample. pH, soil pH; SWC, soil moisture content; EC, soil electric conductivity; SOC, soil organic carbon; AP, soil available phosphorus; TP, soil total phosphorus; TN, soil total nitrogen; NO_3_-N, soil nitrate nitrogen; NH_4_-N, soil ammonium nitrogen; AN, soil alkaline nitrogen.

Principal component analysis (PCA) was employed to investigate the associations between soil physicochemical properties and the level of soil recovery. The analysis of soil property factors at a depth of 0-20 cm in the recovered grassland at the Jinqianghe gold mine indicated that the first axis accounted for 93.22% of the variance, while the second axis explained 2.32%, resulting in a cumulative explanation of 95.55% (**Figure 6A**).At 0-20 cm soil depths, soil properties between HRD and NGL displayed positive correlations with soil water content (SWC), electrical conductivity (EC), available nitrogen (AN), total phosphorus (TP), ammonium nitrogen (NH_4_-N), total nitrogen (TN), available phosphorus (AP), soil organic carbon (SOC), and nitrate nitrogen (NO_3_-N). However, HRD exhibited a negative correlation with soil pH at these depths. At 20-40 cm soil depths, the first axis explained 92.05%, with the corresponding second axis explaining 2.94%, resulting in a cumulative explanation of 94.98% (**Figure 6B**). Soil properties at these depths between HRD and NGL exhibited positive correlations with SWC, AN, TP, NH_4_-N, TN, AP, SOC, and NO_3_-N. Conversely, HRD displayed negative correlations with soil pH and EC at these depths.

## 4 Discussion

### 4.1 Changes in plant community characteristics

To safeguard the vital aspects of plant diversity, nurture plant growth, and effectively monitor restored grasslands, it is imperative to understand the characteristics of plant communities (Catorci and Gratti, 2010; Tang et al., 2016). Many mining areas have undergone restoration using both artificial and natural methods. However, the outcomes have exhibited significant variability, and it has been challenging to distinguish the effects of different mining areas on grassland environments (Huang et al., 2019; Wei et al., 2019). Consequently, field studies are essential to assess and enhance the restoration levels in alpine grasslands. The species composition within plant communities can serve as a reflection of the external appearance of the community, which, in turn, represents the comprehensive influence of environmental factors (Deng et al., 2020). In our study, the vegetation types of restored grassland (VLRD, LRD, MRD, HRD, and NGL) in the mine area were *Carex duriuscula* + *Daucus carota*, *Carex duriuscula* + *Potentilla chinensis*, *Cyperus rotundus* + *Carex duriuscula*, *Carex duriuscula* + *Cyperus rotundus* (**Table 4**). With the degree of restored grassland, the vegetation characteristics showed an increasing trend, the aboveground biomass and coverage increased significantly, and productivity increased (**Figure 2**). Perennial herbaceous plants played the most important role in different restoration degrees of alpine grassland, while annual herbaceous plants were a few plant components (**Table 3**). The plant species dominated by Leguminosae, Asteraceae, Graminaceae, Rosaceae, and Cyperaceae usually grow in low-temperature, high-altitude and alpine grassland (**Table 2**). This is consistent with previous studies that show that these environmental factors preferentially support dominant species’ existence and have poor competition among plant species, and the plant community preserves ecological structure with human intervention (Islam et al., 2022).

The impact of major factors influencing plant value perception remains inconclusive due to variations in study subjects across mine restoration research (Li et al., 2019). The relationships between restoration levels and environmental factors are complex, resulting in diverse plant diversity at different restoration stages (Zhao et al., 2019). Plant diversity serves both as a measure of structural characteristics and an indicator of environmental conditions (Bennett et al., 2020). Prior studies have consistently reported that the importance of plants can directly affect plant diversity (Guo et al., 2020; Hailu et al., 2021). In our findings, the highest number of species was observed in HRD and NGL, whereas VLRD, LRD, and HRD grasslands had *Carex duriuscula* as the dominant species, with corresponding values of 39.22, 27.33, and 18.53, respectively (**Table 4**). The Shannon-Wiener, Simpson, Margalef, and Evenness indexes displayed an upward trend, while the Dominance index exhibited the opposite trend. The HRD Evenness index surpassed that of other restoration levels, possibly influenced by the number of community species and the uniform distribution of individual plants among species. Appropriate replanting and fencing could reduce the likelihood of invasion and enhance other species within the community, consequently promoting community-level plant diversity (**Figure 3**). Previous research has indicated that climate, degree of recovery, precipitation, and human activities like grazing and mining can impact the phenological periods and diversity of vegetation (Bao et al., 2014). Our results affirm that HRD grasslands exhibit richness in species, stable communities, and high uniformity. These findings suggest that HRD can serve as the primary management model for local restoration-level grasslands, providing a critical theoretical foundation for ecological restoration efforts in mining area grasslands. In the case of VLRD, vegetation cover was limited, mainly concentrated on both sides of roads and susceptible to trampling by cattle and sheep. Although fences were initially erected to enclose the restoration area, the large size of the area led to fence destruction by livestock. This necessitates enhanced management measures, including the installation of taller fences, in the later stages of restoration, especially in key secondary restoration areas.

### 4.2 Changes in soil characteristics in different soil layers

Differences in soil fertility as well as geographic location determine differences in vegetation, which in turn absorbs nutrients for plant growth from the soil through nutrients absorbed by the roots into the soil, and to a certain extent plant growth promotes soil nutrients, soil properties play a key role in promoting plant growth, and plant growth can also have an impact on the nutrient properties of the soil (An and Xu, 2013). Among the primary environmental factors affecting plant communities, soil stands out (Yang et al., 2023b). The physical and chemical attributes of soil significantly shape the growth and development of vegetation. In mining grasslands, both soil properties and vegetation growth transform as restoration levels change. Relationships of considerable significance exist among soil characteristics, restoration levels, plant community structure, and plant diversity (Zhang et al., 2020; Huang et al., 2021b). Several studies have shown that restored grasslands could reduce soil pH and EC (Dai et al., 2014, 2017). A similar result was also found in this study, where soil pH and EC were lower from HRD to VLRD due to vegetation growth and sufficient soil nutrients. However, soil SWC, SOC, TN, NO_3_-N, NH_4_-N, AN, TP, and AP were higher from HRD to VLRD (**Figure 4**). Soil pH, EC, and SWC were significantly higher from 20-40 cm soil depths to 0–20 cm soil depths, while soil SOC, TN, NO_3_-N, NH_4_-N, AN, TP, and AP were significantly lower from 20-40 cm soil depths to 0–20 cm soil depths (**Figure 4**). Low-quality soil is apt to cause vegetation degradation (Li et al., 2020), the varying degrees of vegetation coverage could play a crucial role, as vegetation affects soil chemical properties through leaf litter, root exudation, and associations with microorganisms, while an increase in grassland diversity and aboveground biomass leads to further soil restoration (Han et al., 2020). Our results indicate that restored plant communities (VLRD and LRD) had lower plant diversity and aboveground biomass, which might not recover well. Therefore, it is essential to explore plant composition characteristics and soil properties variation in different restoration degrees and take timely policy measures to protect restoration grassland vegetation.

### 4.3 Changes in the correlation between vegetation and soil characteristics

The connection between plant communities and soil properties can vary based on the restoration level, geographic location, and study area (Springate and Kover, 2014; Qin et al., 2019). The results of PCA analysis showed that the composition of the HRD plant community significantly differed from that under VLRD and LRD restoration degrees. The dominant controls for the HRD community composition were soil SWC, EC, AN, TP, NH_4_-N, TN, AP, SOC, and NO_3_-N at soil depths of 0-20 cm and 20-40 cm, respectively. The main reason for this was that the HRD was located at the foot of the mountain, away from the roadside, have sufficient rainfall and was free from human and livestock interference (**Figure 5**). In other studies, soil nutrients have been proven to be the main factor impacting plant growth, distribution and function (Xu et al., 2016, 2019). Soil nutrients exhibited a strong connection with plant communities in both soil depths (Yang et al., 2023b). In the soil properties at 0-20 cm depth, a positive correlation was observed between HRD and NGL with SWC, EC, AN, TP, NH_4_-N, TN, AP, SOC, and NO_3_-N, while HRD exhibited a negative correlation with pH at 0-20 cm soil depths. Similarly, at 20-40 cm depth, the soil properties between HRD and NGL displayed positive correlations with SWC, AN, TP, NH_4_-N, TN, AP, SOC, and NO_3_-N, while HRD showed negative correlations with pH and EC at 20-40 cm soil depths (**Figure 6**). Furthermore, our results confirmed that the HRD restoration degree was similar to NGL, which could enhance the number of vegetation and soil nutrients, leading to long-term mine grasslands restoration. Knowledge on the impact of mining grassland restoration on plant communities and soil properties can provide a scientific basis for the restoration and sustainable utilization of alpine grasslands.

For future restoration of mining areas, an integrated restoration approach should be applied, based on the findings of this study. Specifically, areas with low vegetation cover, classified as VLRD and LRD, should undergo secondary seeding as part of the restoration efforts. Additionally, fencing should be established to prohibit grazing and trampling, taking into account an integrated evaluation of ecological and socio-economic factors. The use of a diverse mixture of native species is recommended to enhance the resilience and stability of the ecosystem. Soil improvement practices, such as the application of organic materials, should be implemented to enhance soil health and fertility, thereby facilitating quicker vegetation establishment and growth. Monitoring and adaptive management practices should involve the implementation of long-term monitoring plans to track restoration progress, with management strategies adjusted as necessary to ensure the effectiveness of the mining area’s restoration efforts.

## 5 Conclusions

This study investigates how environmental factors influence plant community characteristics, plant diversity, and soil properties at various restoration levels. It also aims to identify plant community characteristics along the recovery gradient in the Qilian Mountains study sites. In these sites, we observed 18 species belonging to 11 families and 16 genera across five restoration levels. Notably, *Carex duriuscula*, *Cyperus rotundus*, and *Polygonum viviparum* dominated the areas showing significant recovery, as indicated by their high importance values and species count. Our findings revealed that the Shannon and Simpson indexes in HRD were notably higher compared to VLRD, LRD, and MRD. Moreover, the recovery effect in HRD was similar to that of NGL, demonstrating the most effective natural recovery. Among the environmental factors, soil pH, SWC, SOC, NO_3_-N, and AN played a significant role in regulating the plant community. As restoration levels increased, soil pH and EC gradually decreased, while soil SWC, AN, TP, NH_4_-N, TN, AP, SOC, and NO_3_-N progressively increased. The results suggest that the improvement in grassland restoration is closely linked to soil restoration, particularly the increase in soil SOC and AN content. Furthermore, the enhancement of grassland community characteristics and diversity contributes to further soil restoration. This study establishes a theoretical basis for the restoration and preservation of mining grasslands by revealing the connection between plant community attributes and soil properties in alpine grasslands.

## Data availability statement

The raw data supporting the conclusions of this article will be made available by the authors, without undue reservation.

## Author contributions

XY: Conceptualization, Data curation, Software, Writing – original draft, Writing – review & editing. QF: Funding acquisition, Project administration, Writing – review & editing. MZ: Data curation, Methodology, Software, Writing – original draft. JZ: Investigation, Supervision, Writing – review & editing. LY: Formal analysis, Methodology, Writing – review & editing. CZ: Conceptualization, Validation, Writing – review & editing. ZW: Supervision, Validation, Writing – review & editing. YF: Supervision, Writing – review & editing.
